# Supplementary material for: Global, regional, and country-level cost of leptospirosis due to loss of productivity in humans
Source: PLoS Negl Trop Dis. 2023 Aug 24;17(8):e0011291. doi: 10.1371/journal.pntd.0011291 (PMC10482283; doi:10.1371/journal.pntd.0011291)
Supplement: Attachment — Submitted filename: Reply to Reviewers comments.docx [file pntd.0011291.s002.docx]

**Reply to reviewers comments.**

Reviewer #1:

**I would ask the authors to comment on how they might suggest new study designs to provide the way to the next stage of analysis. For example, how would they present the present analysis to policy makers in LMIC countries where leptospirosis is endemic but the data are sparse?**

Thank you very much for the comment. We have added the suggested way forward to the conclusion paragraph:

Our study emphasises the substantial productivity cost of human leptospirosis, representing only a fraction of the overall economic impact. Estimating the economic impact of a disease requires a comprehensive assessment, considering direct medical costs, productivity losses due to absenteeism and reduced labour efficiency, increased healthcare spending, and potential disruptions in supply chains and business operations. The main issue in the estimation of the economic burden of leptospirosis is the lack of primary data sources. The scarcity of field-based cost-of-illness (COI) studies will hamper the accuracy of any modelling exercises (econometrics, statistical models). We agree that it poses a significant challenge to the accuracy of modelling exercises, making it essential to urgently incorporate the COI assessment into field studies to comprehensively understand the global burden of leptospirosis. In addition to economic considerations, a thorough understanding of the disease epidemiology is essential to elucidate transmission patterns, affected population demographics, and potential long-term effects on workforce health. These considerations highlight the need for increasing the number of primary data points as the next step. Anticipated projections suggest an increase in leptospirosis cases attributable to unplanned urbanisation and climate change, demanding urgent action through a comprehensive One Health approach that integrates both animal and human health. Policymakers and health organisations are urged to prioritise leptospirosis as a Neglected Tropical Disease and allocate resources towards the development and implementation of effective prevention and control measures, including vaccine development.

These considerations have presented in the paragraph of the conclusion of the manuscript.

Reviewer #2:

**Please provide further elaboration in the text manuscript about the concept of international dollars (Int$) in comparison to USD for better understanding and clarification.**

Thank you very much for the suggestion. We added the following description to the methods section of the manuscript:

The Int$, also known as the Geary-Khamis dollar, is a hypothetical unit of currency that has the same purchasing power parity that the US dollar has in the United States at a given point in time. It allows for comparing the purchasing power parity between countries. In other words, Int$ follows the law of one price, reflecting people’s living standards comparably across countries. It is widely employed by the International Comparison Program and the World Bank for international economic analysis and comparisons.

These explanations have been provided in the manuscript.
